# Critical Point Drying: An Effective Drying Method for Direct Measurement of the Surface Area of a Pretreated Cellulosic Biomass

**DOI:** 10.3390/polym10060676

**Published:** 2018-06-17

**Authors:** Kyu-Young Kang, Kyung-Ran Hwang, Ji-Yeon Park, Joon-Pyo Lee, Jun-Seok Kim, Jin-Suk Lee

**Affiliations:** 1 Department of Biological and Environmental Science, Dongguk University-Seoul, 32 Dongguk-ro, Ilsandong-gu, Goyang 10326, Korea; kykang@dongguk.edu; 2 Biomass and Waste Energy Laboratory, Korea Institute of Energy Research, 152 Gajeong-ro, Yuseong-gu, Daejeon 34129, Korea; hkran@kier.re.kr (K.-R.H.); yearn@kier.re.kr (J.-Y.P.); 3 Gwangju Bioenergy R&D Center, Korea Institute of Energy Research, 25 Samso-ro270beongil, Buk-gu, Gwangju 61003, Korea; bmjplee@kier.re.kr; 4 Department of Chemical Engineering, Kyonggi University, 154-42 Gwanggyosan-ro, Yeongtong-gu, Suwon 16227, Korea; jskim84@kyonggi.ac.kr

**Keywords:** pretreated cellulosic biomass, critical point drying, surface area, pore size distribution, Brunauer–Emmett–Teller (BET), cellulose, hornification

## Abstract

The surface area and pore size distribution of *Eucalyptus* samples that were pretreated by different methods were determined by the Brunauer–Emmett–Teller (BET) technique. Three methods were applied to prepare cellulosic biomass samples for the BET measurements, air, freeze, and critical point drying (CPD). The air and freeze drying caused a severe collapse of the biomass pore structures, but the CPD effectively preserved the biomass morphology. The surface area of the CPD prepared *Eucalyptus* samples were determined to be 58–161 m^2^/g, whereas the air and freeze dried samples were 0.5–1.3 and 1.0–2.4 m^2^/g, respectively. The average pore diameter of the CPD prepared *Eucalyptus* samples were 61–70 Å. The CPD preserved the *Eucalyptus* sample morphology by replacing water with a non-polar solvent, CO_2_ fluid, which prevented hydrogen bond reformation in the cellulose.

## 1. Introduction

For the efficient sugar fractionation from the lignocellulosic biomass, physical contact between cellulose and cellulase enzymes is necessary. Therefore, the cellulose specific surface area that is available for enzyme contact is one of the most important factors for determining the rate and extent of the enzymatic hydrolysis of the biomass [1,2,3]. Since the average size of cellulase enzymes is approximately 5.1 nm, the internal surface of the pores that are greater than 5.1 nm should be particularly effective for enzymatic hydrolysis [4]. Various pretreatments that have been applied for the improved enzymatic digestibility also increase the surface area, as a result of not only the removal of hemicellulose and/or lignin, but also cellulose swelling. Most studies regarding the biomass specific surface area and pore size distribution have employed indirect measurement techniques, such as solute exclusion [5,6,7,8], non-hydrolytic protein adsorption [9], Simons’ staining [10,11,12], and NMR techniques [13,14].

Solute exclusion is the most widely employed method, but it has several drawbacks, including a relatively low accuracy and limitations on the pore size ranges that can be determined. For example, given the unavailability of dextran molecule probes, only pore sizes up to 56 nm can be measured [5,6,7,15]. However, the non-ionic surfactant pretreatment of the lignocellulosic biomass can produce pores with up to a 100 nm diameter [16]. Inaccurate estimation can also arise from the water competition with the solute probes [1] and/or solute concentration measurement errors. The reported biomass surface area varies greatly, from 20 to over 1500 m^2^/g [7,15].

The Simons’ staining method has also been used to determine the feasibility of the enzymatic hydrolysis of substrates [10,11,12,17], although this technique can provide only semi-quantitative information. It also has similar limitations to the solute exclusion, since it also employs dye solutes for the measurement. On the other hand, the NMR techniques that have been employed for the biomass surface area measurement require complicated experiment set-ups [13,14].

The Brunauer–Emmett–Teller (BET) technique employing N_2_ adsorption has many advantages, including a high accuracy, and it can measure 0.4–300 nm pore sizes [18]. Therefore, the BET technique is widely used to determine the surface area and pore size distribution of porous materials, although it is applicable only to dried samples. Few studies have considered the BET based internal surface area measurement of the pretreated biomass [11,19,20]. However, these have indicated that the BET measured surface areas differ significantly from those that have been measured by other methods, (i.e., solute exclusion, dye staining, or probes). For example, Wiman et al. compared a steam pretreated spruce surface area using the BET and Simons’ staining methods [11]. The BET measurement biomass samples were oven dried at 30 °C for 24 h to minimize structural changes. The pretreated biomass surface area was 1.3–8.2 m^2^/g, far smaller than those that were measured by the staining method (53–64 m^2^/g). The small BET based surface area was attributed mainly to the pore collapse during air drying [12,21].

To avoid a pore collapse, freeze drying has been applied to cellulose and yellow poplar (*Liriodendron tulipifera*) wood flour, which has been pretreated by the organosolv process [19,20], but the biomass surface area remains small, 5–39 and 1.8 m^2^/g for thecellulose and organosolv pretreated yellow poplar, respectively. Esteghalian et al. investigated the effects of the drying conditions on the enzymatic hydrolysis of Douglas fir (*Pseudotsuga menziesii*) kraft pulp using air, oven, and freeze drying; and compared the dried biomass enzymatic digestibilities. No significant differences between the air and freeze dried biomass samples were evident [17]. Thus, no successful method remains that prevents pore collapse when drying the cellulosic biomass. Therefore, the cellulosic biomass surface area is mainly determined by in situ measuring techniques.

Critical point drying (CPD) is widely used to dry delicate samples for scanning electron microscope (SEM) applications and could be a viable option for biomass sample preparation, while maintaining the original morphology. Since pore collapse is caused by the removal of water (a polar solvent) from the biomass [12,21], this study attempted to prevent pore collapse by replacing water with non-polar solvents before drying. Since CPD employing a non-polar solvent (liquid CO_2_) is done at ~36 °C, deterioration that is caused by a high drying temperature can be minimized. There have been no previous reports on the direct measurement of the surface area and/or pore size distribution for the CPD pretreated cellulosic biomass.

This study prepared *Eucalyptus* wood flour samples using three pretreatment methods to measure the total surface area and pore size distribution, and the drying conditions’ effects on the surface area and pore size distribution were compared. This work will contribute to a deeper understanding of the physical effects of surface area and pore size distribution on enzymatic hydrolysis rates of cellulosic biomass.

## 2. Materials and Methods

### 2.1. Materials

The *Eucalyptus* (*E. grandis*) wood chips were supplied by Dr. Zhuang of the GuangZhou Institute of Energy Conversion, Chinese Academy of Science (GIEC) in China; knife milled by the Wiley mill (Mini Wiley mill, Thomas Scientific, Swedesboro, NJ, USA); and screened to a nominal size of 20–60 mesh. The alkaline electrolyzed water (ALEW) was provided by Gendocs Inc. (Daejeon, Korea) with a pH = 12.2 and ORP < −795 mV. All of the other reagents and chemicals were of an analytical grade and were purchased from either Sigma-Aldrich (St. Louis, MO, USA) or local suppliers in Korea.

### 2.2. Pretreatments of Lignocellulosic Biomass

The *Eucalyptus* samples as a lignocellulosic biomass were pretreated by dilute acid (DA), steamed after NaOH impregnation, and ALEW. For the DA pretreatment, 60 g (OD) of a biomass sample was immersed in 160 mL of 3% (*w*/*w*) sulfuric acid and was maintained at 121 °C for 2 h. The slurry was allowed to stand overnight and was then filtered (Whatman No. 1 glass filter) to recover the insoluble solids. The recovered cellulosic biomass was washed with distilled water several times. The process of NaOH–steam pretreatment followed the procedure that was detailed previously by the authors of [22]. Then, 60 g (OD) of the biomass sample was soaked in 480 mL of s 3% (*w*/*v*) sodium hydroxide solution at room temperature. The slurry was allowed to stand overnight and was then filtered (Whatman No. 1 glass filter) to recover the insoluble solids. The recovered solids were transferred to an autoclave (working volume = 1 L) and the steam pretreatment was conducted at 160 °C for 12 min under a 20 bar nitrogen atmosphere. For the ALEW pretreatment, 60 g (OD) of the biomass sample was immersed in 600 mL ALEW and was maintained at 180 °C for 1 h in an autoclave (working volume = 1 L) under a 20 bar nitrogen atmosphere.

### 2.3. Drying of Cellulosic Biomass Sample

The pretreated samples were dried by air, were frozen, and by critical point drying methods. For the air drying (AD), approximately 5 g of each of the pretreated cellulosic biomass samples were dried in a vacuum oven at 50 °C for 48 h. The constant weight was confirmed after drying. The freeze drying (FD) process of the cellulosic biomass sample followed that which was detailed in the literature [19]. Approximately 5 g of the pretreated cellulosic biomass sample was frozen at −60 °C for 48 h, and was then vacuumed in a FD apparatus for 72 h. The critical point drying (CPD) was performed using a critical point dryer (13200J-AB, SPI Supplies, West Chester, PA, USA), following the procedure that was provided in the operation manual. Approximately 5 g of the pretreated biomass samples were placed in 100 mL of 30, 50, 70, 90, 95, and 100% ethanol for 15 min, and were then immersed in an acetone solution for a further 15 min. After a series of the solvent exchanges, the acetone in the samples became replaced by liquid CO_2_, and the samples were then critical point dried at 36 °C.

### 2.4. Surface Area and Pore Size Measurements of Biomass Sample

We used the BET method to determine the surface area, average pore diameter, and total pore volume for the AD, FD, and CPD biomass samples. The N_2_ adsorption was measured using an accelerated surface area and porosity analyzer (ASAP 2420, Micromeritics Inc., Norcross, GA, USA). The N_2_ adsorption isotherms were obtained by measuring the amount of gas that was adsorbed across a range of relative pressures (*P*/*P_0_*) at constant temperature (−196 °C, liquid nitrogen phase temperature), where *P* and *P_0_* were the equilibrium and saturation pressures, respectively, of the adsorbate gas at the temperature of adsorption. The desorption isotherms were achieved by measuring the amount of N_2_ gas that was removed as the pressure decreased. Subsequently, the specific surface area was calculated from the adsorption isotherms, using the BET theory. The total pore volume was estimated from the amount of N_2_ gas that was adsorbed at 0.98 of the relative pressure, under the following assumptions: the pores were filled with liquid nitrogen, and adsorption average pore size was derived from 4*V*/*A*, where *V* was the total pore volume, and *A* was the surface area corresponding to the assumed cylindrical pore model. The pore size distribution was obtained from the experimental isotherms, as detailed elsewhere [23]. Prior to the BET analysis, the pre-dried samples were degassed at 90 °C for 0.5 h, and then again at 105 °C for 4 h.

### 2.5. FTIR and SEM Analyses

The structural changes of the raw and pretreated samples were examined with Fourier transform infrared spectroscopy (FTIR). The samples were ground into a powder and sieved through a 149 μm mesh. The FTIR spectra were recorded on an FTS-175C (Bio-Rad Laboratories Inc., Hercules, CA, USA) that was equipped with a mercury cadmium telluride detector, using KBr pellets. All of the spectra were collected at a 4 cm^−1^ resolution, with 32 scans in the range 4000–500 cm^−1^. The SEM observation was also performed to confirm the structural changes of the samples using the FE-SEM (S-4700, Hitachi, Tokyo, Japan).

## 3. Results and Discussion

### 3.1. Drying Methods Effects on Surface Area and Pore Size Distribution of Pretreated Eucalyptus

**Figure 1:**
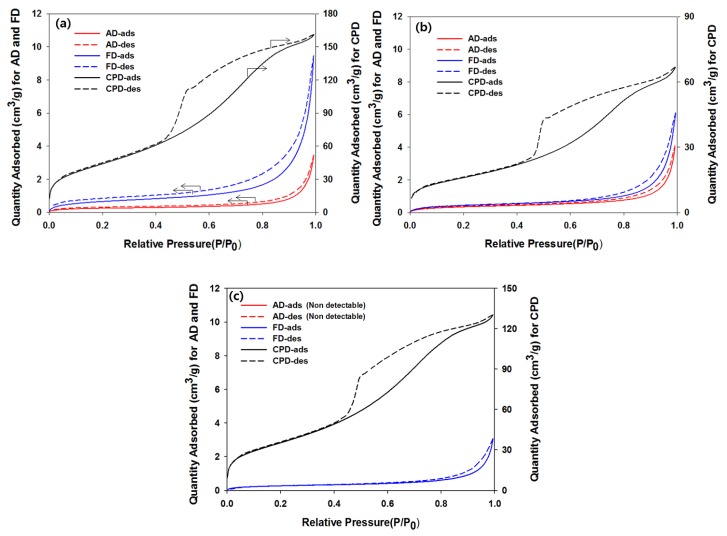
Nitrogen adsorption–desorption isotherms for air (AD), freeze (FD), and critical point (CPD) dried *Eucalyptus* samples with (**a**) ALEW, (**b**) 1% H_2_SO_4_, and (**c**) NaOH–steam pretreatments.

The surface area and pore size distribution of the samples were determined by BET and the differences were compared among the CPD, AD, and FD pretreatments. Figure 1 shows the N_2_ adsorption–desorption isotherms for ALEW, DA, and NaOH–steam pretreated samples. The isotherms were the typical type IV hysteresis loops (as classified by IUPAC), which were consistent with mesoporous materials, where an adsorbate monolayer was formed on the pore surface at low pressures, followed by a multilayer formation. The hysteresis loop originated from the capillary condensation in meso- and macro-pores, and could have a wide variety of shapes, depending on the pore geometries. Specific pore structures could often be identified from their hysteresis loop shape, based on the empirical IUPAC classification. The AD and FD samples also exhibited H4 type hysteresis loops, although somewhat smaller. This hysteresis type was consistent with narrow slit shaped pores and/or aggregated particles [24]. The pore collapse in the cellulose fibers was more significant during the AD procedure than the FD, based on their adsorbed quantity. All of the CPD samples exhibited H2 type hysteresis loops, where the pore size and pore shape distribution were difficult to interpret well. According to the literature [25,26,27], the H2 loop with the steeper desorption slope than the adsorption slope occurred in the network of the ink-bottle pores. This meant that the cellulose fibers cell walls were partially collapsed and then the narrow pore necks and the wide pore body were interconnected during the CPD procedure.

**Figure 2:**
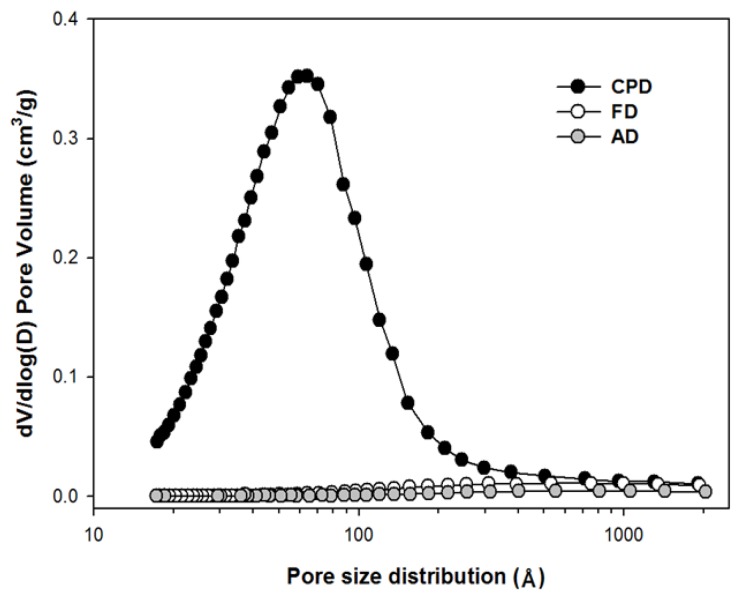
Drying method effects on pore size distribution for alkaline electrolyzed water (ALEW) pretreated *Eucalyptus* samples.

Figure 2 shows the corresponding biomass pore size distribution, which was determined by the BJH (Barrett–Joyner–Halenda) method. As reported elsewhere, the AD and FD samples’ large pores indicated a collapse of most of the small pore structures during drying [25]. However, the CPD showed distinguishable pore volume and appeared to maintain the pore structures in the pretreated *Eucalyptus* samples, with the average pore size of approximately 6.2 nm (i.e., within the mesopore range).

**Figure 3:**
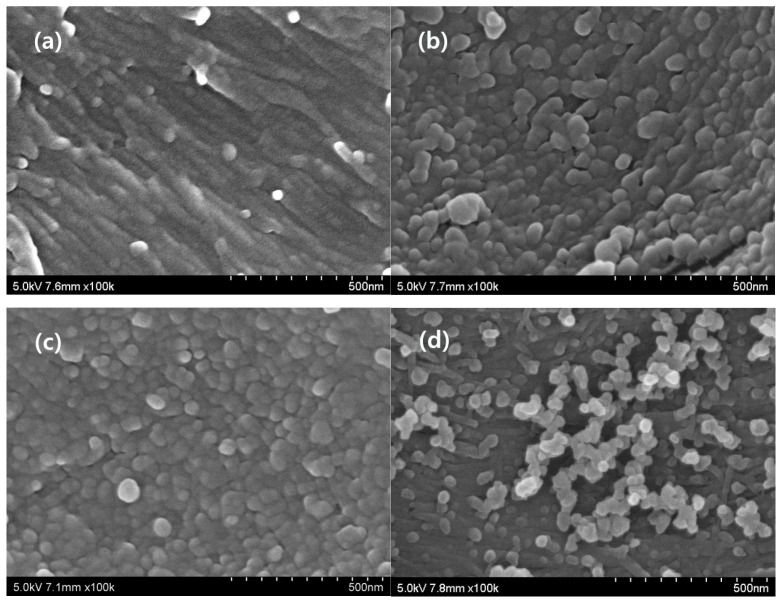
SEM images of the ALEW pretreated *Eucalyptus* samples, according to the different drying methods, namely: the (**a**) untreated control, (**b**) air drying (AD), (**c**) freeze drying (FD), and (**d**) critical point drying (CPD).

Table 1 shows the detailed quantitative data for the ALEW pretreated *Eucalyptus* samples. The surface area of the pretreated *Eucalyptus* samples varied greatly with the drying conditions. The smallest surface areas were from the AD samples (0.5–1.3 m^2^/g), with the FD samples having approximately twice the area (1.04–2.44 m^2^/g), although they had comparable small pore volumes (0.002–0.005 and 0.005–0.015 cm^3^/g, respectively); whereas the CPD samples’ surface area and pore volume were considerably larger (58.5–161.5 m^2^/g and 0.103–0.249 cm^3^/g, respectively). The structural changes of the ALEW pretreated *Eucalyptus* samples, according to the different drying methods, were confirmed by the SEM observation, as shown in Figure 3. From the results of the SEM observations, it was obvious that the CPD method preserved the microstructure and was effective in the drying of the pretreated cellulosic biomass samples. It was a similar result to the study on the application of the CPD for the accurate surface area measurement of the other samples [28].

As shown in Table 2, the surface area of the AD *Eucalyptus* samples was somewhat smaller than what was previously reported for SO_2_–steam pretreated spruce (1.3–8.2 m^2^/g) [11], which was possibly because of the different feedstocks and pretreatment conditions. However, the surface area of the SO_2_–steam pretreated spruce varied greatly with the pretreatment conditions that were tested in that study. The surface area of the FD *Eucalyptus* samples was comparable to that which was previously reported for the yellow poplar (*L. tulipifera*) (1.80 m^2^/g) and were prepared by FD after the organosolv pretreatment [20]. Table 3 compares the surface areas of the CPD *Eucalyptus* samples with those that were previously reported. The CPD surface area for the DA pretreated *Eucalyptus* (57.3 m^2^/g) was very close to the SO_2_–steam pretreated spruce that was determined by dye staining (58.5 m^2^/g) [11].

Thus, only the CPD effectively maintained the pretreated biomass morphology, although both of the FD and CPD were previously reported as effective. Both of the drying methods were widely employed in the SEM specimen preparation [29]. The poor performance of the FD for the water-swollen lignocellulose that were reported here was probably associated with the cellulose content of the feedstock, since the cellulose was deformed by hornification, a consequence of the irreversible changes to the cell wall structure [12].

**Figure 4:**
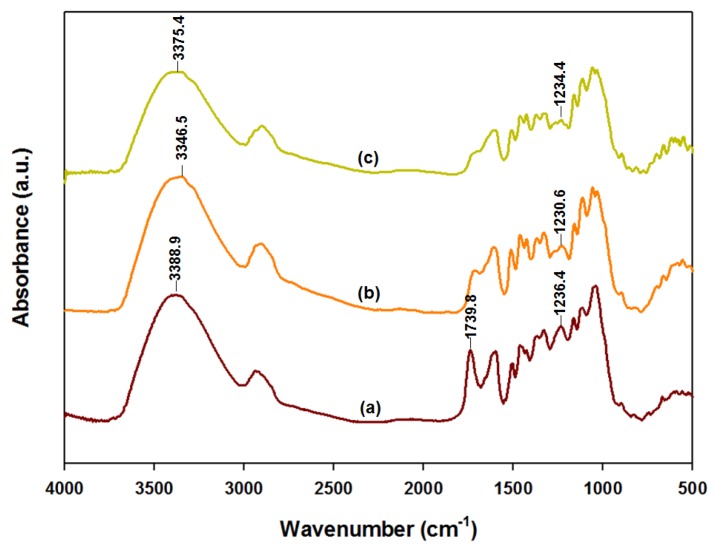
FTIR spectra for the dried *Eucalyptus* samples, before and after ALEW, as follows: (a) untreated control, (b) air drying (AD) after ALEW, and (c) critical point drying (CPD) after ALEW.

Although the exact hornification mechanism was unclear, one possible explanation was the hydrogen bond breaking and reforming, corresponding to the cellulose wetting and drying, respectively. When the cellulose was wet, the fibers swelled by the hydrogen bond breakage, but shrunk with the reforming of the hydrogen bonds upon drying. In any case, the reason for the poor FD performance remained unknown. Possibly FD removed only free water, but not the bound water, inducing the cellulosic collapse [30]. However, hydrogen reforming could be prevented if the bound water in the pretreated *Eucalyptus* samples was replaced by a non-polar solvent prior to drying. To examine this hypothesis, we performed an FTIR analysis to examine the hydrogen bonds in the ALEW pretreated AD and CPD *Eucalyptus* samples, as shown in Figure 4.

The O–H alcohol stretching region of the FTIR absorbance band, 4000–3000 cm^−1^, was reported to contain the information on hydrogen bonding in cellulose [31,32], and large peaks were observed between 3390–3340 cm^−1^. The ALEW pretreated CPD *Eucalyptus* sample peaks were significantly smaller than those of the untreated and ALEW pretreated AD *Eucalyptus*. The AD *Eucalyptus* peak height was very similar to that of the untreated *Eucalyptus*. Thus, since the peak height was proportional to the hydrogen bonds in the biomass sample, the CPD *Eucalyptus* had significantly fewer hydrogen bonds than the untreated or AD *Eucalyptus*. The peaks at 1739.8 cm^−1^ and between 1260–1234 cm^−1^ indicated C=O carbonyls and O–H phenolic, respectively, and these peaks were getting smaller by drying. Overall, the hydrogen bonds in the untreated *Eucalyptus* were broken by water during the pretreatment and were subsequently reconnected upon the AD. However, the hydrogen bond reformation upon drying was successfully prevented for the CPD *Eucalyptus* samples, replacing the bound water with a non-polar solvent (liquid CO_2_), prior to drying. Finally, the wide peak at 3388.9 cm^−1^ shifted to s higher frequency after the drying of the ALEW pretreated *Eucalyptus*. This shift was more significant with the AD, where the hydrogen bonding became stronger than what was the case for the untreated sample.

### 3.2. Pretreatment Conditions Effects on Surface Area and Pore Size Distribution

**Figure 5:**
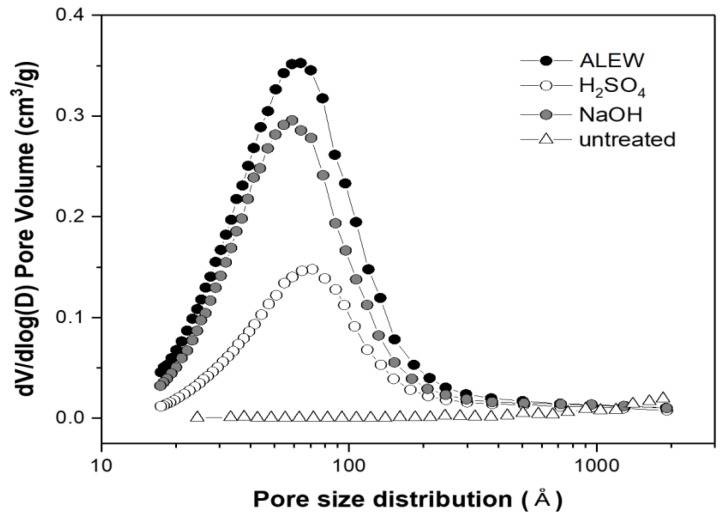
Pretreatment method effects on the pore size distribution for the ALEW critical point dried *Eucalyptus* samples.

Since the pore volumes varied sharply with the pretreatment methods (Figure 5), we investigated the pretreatment condition influences on the pore volumes, surface area, and pore size distribution for the CPD *Eucalyptus* samples. The alkali pretreatment yielded a biomass with a higher pore volume and surface area than those that were achieved by the acid pretreatment. The swelling effects of the alkali pretreatment have been reported previously. Huang et al. showed that NaOH pretreated corn cob had an approximately 40% larger surface area (57.4 m^2^/g) than the corresponding sulfuric acid pretreated sample [7]. In the current study, the ALEW pretreatment exhibited the strongest swelling effect on the *Eucalyptus* samples, with an approximately 20% higher pore volume but similar average pore size, hence, an approximately 20% larger surface area than the NaOH–steam pretreatment.

Table 4 summarizes the effects of these and the other quantified pretreatments on the pore volume and surface area of the *Eucalyptus* samples. The DA pretreatment exhibited the lowest pore volume and the smallest surface area, approximately 45% of the values from the NaOH pretreated *Eucalyptus* samples. The reason for the smaller surface area with the DA pretreatment was unclear, although it was likely because of cellulose aggregation. Changes in the chemical composition could also be another possible reason, as the removal of the lignin and hemicellulose had a significant effect on the surface area and pore size distribution [7].

The biomass pore volume and surface area provided the enzymes with sufficient access and adsorption to the cell surfaces, thus significantly affecting the enzymatic hydrolysis of the cellulose. Therefore, we determined the pore volumes for the different pore sizes, as shown in Table 5. The ALEW pretreated *Eucalyptus* samples exhibited the largest pore volume accessible to the enzymes, with the NaOH pretreated samples exhibiting the highest pore volumes for the pores that were smaller than 2 nm, and the ALEW pretreated samples had the highest pore volumes for the larger pore sizes. The 25% larger surface area of the ALEW pretreated samples was remarkable, even after considering the higher pore volume for the larger than 2 nm pores. This larger surface area was probably because of the high lignin content, since most of the lignin micropores were smaller than 0.6 nm [34].

## 4. Conclusions

Critical point drying was shown to effectively prevent cellulosic pore collapse upon drying, and hence enabled the direct determination of the specific surface area and pore size distribution for pretreated the *Eucalyptus* samples using the BET method. Comparing hydrogen bonds for the various drying methods, the reformation of hydrogen bonds upon drying is mainly responsible for pore collapse. Thus, hydrogen bond reformation was successfully prevented in CPD by replacing the water with liquid CO_2_ (a non-polar solvent) before drying.

The measurement technology that was developed in this study will provide more detailed quantitative data on the surface area and pore size distribution of the water-swollen biomass.

The surface areas of the CPD *Eucalyptus* samples were 58–161 m^2^/g, comparable to those that were determined by the indirect measuring methods; whereas the sulfuric acid pretreatment yielded a considerably smaller surface area with a larger average pore diameter, the ALEW pretreatment produced the highest surface area, and the NaOH–steam pretreatment produced a somewhat smaller surface area.

## Figures and Tables

**Table 1:** Mean surface areas, pore diameters, and total pore volumes for the alkaline electrolyzed water (ALEW) pretreated *Eucalyptus* samples.

Pretreatment	Drying Method	Surface Area (m^2^/g)	Average Pore Diameter (Å)	Total Pore Volume (cm^3^/g)
ALEW	AD ^1^	0.9	–	0.005
	FD ^2^	2.4	–	0.015
	CPD ^3^	161.5	61.7	0.249

^1^ Air drying. ^2^ Freeze drying. ^3^ Critical point drying.

**Table 2:** Drying method effect on the Brunauer–Emmett–Teller (BET) surface area.

Biomass	Drying Condition	Surface Area (m^2^/g)	Reference
Spruce ^1^	Air drying at 30 °C	6.3	[11]
Yellow poplar ^2^	Freeze drying	1.8	[20]
*Eucalyptus* ^3^	Critical point drying	58.5	Current paper

^1^ Pretreated by 2.5% SO_2_ at 207 °C for 7 min. ^2^ Pretreated by EtOH (organosolv) at 130 °C for 50 min. ^3^ Pretreated by 1% H_2_SO_4_ at 121 °C for 2 h.

**Table 3:** Comparison of the BET results with those that were determined by non-drying measurement techniques.

Biomass	Measurement Technique	Surface Area (m^2^/g)	Surface Area Accessible to Cellulose (m^2^/g)	Reference
Corn cob	Solute exclusion	–	57.4 ^1^ 34.1 ^2^	[7]
	Protein adsorption	–	7.7 ^3^	[33]
Lodgepole pine pulp ^4^	Solute exclusion	–	22.4	[8]
Mixed hard wood ^5^	Solute exclusion	1134	25.8	[15]
Filter paper	Protein adsorption	–	9.8	[9]
Spruce ^6^	Dye staining	57.3	–	[11]
*Eucalyptus* ^7^	BET	58.5	–	Current paper

^1^ Pretreated by 2% NaOH at 80 °C for 6 h. ^2^ Pretreated by 2% H_2_SO_4_ at 120 °C for 0.75 h. ^3^ Pretreated by 0.048 g H_2_SO_4_/g biomass at 190 °C for 1 min. ^4^ Pretreated by SPORL at 180 °C for 20 min. ^5^ Pretreated by 1% H_2_SO_4_ at 180 °C for 8.3 s. ^6^ Pretreated by 2.5% SO_2_ at 207 °C for 7 min. ^7^ Pretreated by 1% H_2_SO_4_ at 121 °C for 2 h.

**Table 4:** Effects of the pretreatment conditions on the surface areas, average pore sizes, and pore volumes of the critical point drying (CPD) *Eucalyptus* samples.

Pretreatment	Surface Area (m^2^/g)	Average Pore Diameter (Å)	Total Pore Volume (cm^3^/g)
Untreated	0.8	34.1	0.007
ALEW ^1^	161.5	61.7	0.249
DA ^2^	58.5	70.6	0.103
NaOH–steam ^3^	129.9	62.1	0.202

^1^ Pretreated by alkali electrolyzed water (ALEW) at 180 °C for 1 h. ^2^ Pretreated by 1% H_2_SO_4_ at 121 °C for 2 h. ^3^ Pretreated by steam at 160 °C for 12 min after 3% NaOH soaking for 12 h. DA—dilute acid.

**Table 5:** Effects of pretreatment conditions on micropore volumes of the *Eucalyptus* samples.

Pretreatment	Pore Diameter (nm)			
	*P_d_* < 2	2 < *P_d_* < 5	5 < *P_d_*	Total
ALEW	0.019	0.08	0.15 (60%) *	0.249
1% H_2_SO_4_	0.003	0.03	0.07 (68%)	0.103
NaOH–steam	0.022	0.06	0.12 (59%)	0.202

* Pore volume fraction.
